# Development of a DNA Barcoding-Like Approach to Detect Mustard Allergens in Wheat Flours

**DOI:** 10.3390/genes10030234

**Published:** 2019-03-19

**Authors:** Jessica Frigerio, Roberta Pellesi, Valerio Mezzasalma, Fabrizio De Mattia, Andrea Galimberti, Francesca Lambertini, Michele Suman, Sandro Zanardi, Andrea Leporati, Massimo Labra

**Affiliations:** 1 FEM2-Ambiente, Piazza della Scienza 2, I-20126 Milano, Italy; jessica.frigerio@fem2ambiente.com (J.F.); valerio.mezzasalma@fem2ambiente.com (V.M.); fabrizio.demattia@fem2ambiente.com (F.D.M.); 2 BARILLA G. e R. FRATELLI Spa, Via Mantova, 166-43122 Parma, Italy; roberta.pellesi@barilla.com (R.P.); francesca.lambertini@barilla.com (F.L.); michele.suman@barilla.com (M.S.); sandro.zanardi@barilla.com (S.Z.); andrea.leporati@barilla.com (A.L.); 3 Zooplantlab, Department of Biotechnology and Biosciences, University of Milano-Bicocca, Piazza della Scienza 2, I-20126 Milano, Italy; andrea.galimberti@unimib.it

**Keywords:** allergen detection, DNA barcoding, Brassicaceae, *Brassica napus*, processed food, *Triticum aestivum*

## Abstract

The spread of food allergens is a topic of global importance due to its impact on public health. National and International regulations ask food producers and manufacturers to declare product compositions on the label, especially in case of processed raw materials. Wheat flour (*Triticum aestivum*) can be contaminated by a wide range of species belonging to the Brassicaceae in the field or during grain harvests, storage, and processing. Among them, mustards (*Brassica nigra*, *Brassica juncea* and *Sinapis alba*) are well known allergenic species. Often, food quality laboratories adopt an ELISA approach to detect the presence of mustard species. However, this approach shows cross-reactivity with other non-allergenic species such as *Brassica napus* (rapeseed). In the last few years, DNA barcoding was proposed as a valid identification method, and it is now commonly used in the authentication of food products. This study aims to set up an easy and rapid DNA-based tool to detect mustard allergenic species. DNA barcoding (*matK* and ITS2) and chromosome markers (A6, B, C1 genome regions) were selected, and specific primers were validated on incurred reference food matrices. The developed test was proven to be able to distinguish mustard from rapeseed and wheat, overcoming cross-reactivity with *Brassica napus*.

## 1. Introduction

Food allergens are among the most pressing concerns regarding global public health. Exposure to low doses of an allergen can provoke a severe response in sensitive persons, and the only effective way to prevent this is to avoid food items that may contain such allergens. The most recent European Directive concerning food safety (No. 1169/2011) lists 14 main allergenic food ingredients. Food producers and manufacturers have to be sure of the absence of these compounds and declare products as safe on the label [1]. It is important to highlight that unintentional “cross-contact” between different food ingredients with allergens can occur not only in raw materials but also during processing, in reworked products, or due to allergen carry-over from the use of shared equipment or production plants [2]. To prevent cross-contamination in terms of allergenic substances between food products, many food manufacturers put in place very strict allergen-control measures, such as the use of dedicated facilities or production lines.

Although there are legal indications on how to declare allergens in food, globalization has certainly increased the number of proteins that can be found in foods and are able to cause allergic reactions in sensitive subjects [3]. Therefore, food safety strategies demand innovative analytical methods to control raw materials and processed food products to verify the absence of known and unknown contaminants. Omics technologies offer a suitable solution [4]. Proteomics arguably represent the best option to identify new allergenic proteins and, in combination with immunoassays, they offer the opportunity of detecting allergenic compounds. On the other hand, DNA-based methods have been developed over recent decades to identify and quantify allergenic food components [5]. Polymerase Chain Reaction techniques (PCR) and related approaches have also been applied to the detection of genome fragments encoding allergenic food proteins, and have progressively been implemented in routine analyses for semi-quantitative/quantitative analyses of food allergens [5,6].

Among the PCR-based methods, DNA barcoding has largely been used to identify the taxonomic identity of raw and transformed food materials [7,8]. This technique, which is based on the amplification and sequencing of universal genome region(s), shows several important advantages. First of all, due to the availability of comprehensive reference sequence libraries (e.g., the barcode of life database BOLD and NCBI GenBank), the DNA barcoding approach is able to identify any kind of organism (even those not listed on a putative label) occurring in the analyzed food item. Moreover, it is a relatively easy and low-cost analytical method, suitable for routine analysis in industrial food companies. The success of DNA barcoding has progressively been recognized by governmental authorities, which proposed its official adoption for the authentication of some food categories, as was the case of fish-based products by the US FDA [9]. Moreover, new regulatory directives concerning food labeling, such as the European Regulation No. 1169/2011 [1] on the provision of food information to consumers, will inevitably drive national institutions towards the use of molecular DNA-based tools to address the issues of food authenticity and safety. Several papers suggest the efficacy of the DNA barcoding approach in complementing food quality and safety control activities [7,10]. In general, this tool has a higher efficacy when the investigated species show a great interspecific divergence and a smaller intraspecific one. This is strictly related to the phylogenetic relationships between the contaminant taxa and the declared food ingredient species [11,12]. For example, Federici and co-workers [11] showed that DNA barcoding does not distinguish between some cryptic taxa of Lamiaceae (e.g., Thymus) due to their low phylogenetic structure or to introgression events among different morphospecies. Conversely, other studies supported a clear gap between the two sources of genetic variability (i.e., intraspecific vs. interspecific) that were able to distinguish both animal and plant food raw materials [12].

In this study we investigated common wheat flour (*Triticum aestivum* L.) contamination by species belonging to the Brassicaceae family (e.g., radish, various types of cabbage, broccoli, turnip, rapeseed, and many others). Most of these contain allergenic elements [13]; specifically, we focused on those plants related to mustard, i.e., *Sinapis alba* L. (yellow mustard, or white mustard), *Brassica juncea* L. Czern. (also called brown mustard or Indian mustard), and *Brassica nigra* L. (Black mustard). These species contain the allergenic 2S albumin, which can produce serious health reactions in sensitive subjects [14]. Therefore, the European Regulation 1169/2011 established that producers have to declare the presence of such compounds on the label of their products. Currently, laboratories working in the food sector often adopt an ELISA approach [15] to detect possible food contamination by mustard species. However, this approach could give false positive results due to the frequent cross reactivity with other non-allergenic species such as *Brassica napus* L. (rapeseed) [16]. Moreover, allergic reactions to rapeseed have never been described in mustard-allergic patients [17]. In addition, *B. napus* is the second most important oilseed crop of the world and, in agricultural production, a very suitable complement for rotations with cereals like wheat [18].

The work here presented aims to set up a reliable DNA-based tool to detect mustard allergenic species which is able to meet food companies’ needs. The DNA test should be able to identify mustard species and exclude the false positivity with *B. napus* in wheat flour, avoiding erroneous food allergen labeling, and the loss or discarding of food products.

## 2. Materials and Methods

### 2.1. Reference Plant Dataset

To assess the efficacy of a DNA-based approach to identify the allergenic taxa belonging to the Brassicaceae family, three geographically different seed batches of the most allergenic mustard species (i.e., *S. alba*, *B. juncea,* and *B. nigra*) and the non-allergenic *B. napus* were sourced from the Centre of Genetic Resources (CGN, Wageningen, The Netherlands) (Table S1). Additionally, DNA of *T. aestivum* was obtained from uncontaminated wheat flour.

### 2.2. Reference Common Wheat Flour Matrices Preparation

Starting from a 20 kg batch of common Italian grains (i.e., *T. aestivum*), a homogeneous wheat flour was obtained in a pilot plant, according to common milling procedure. Water was added to the wheat and the mixture was left at room temperature for 36 h in order to let the bran separate from the grain of wheat. This step allowed the moisture level to reach 16.50% (starting from 11.30%), improving the division of husks from wheat during grinding. A Bona mill (Bona S.r.L., Italy) with a capacity of 1–4 kg/h was used. This lab scale mill can simulate the industrial milling process and separate the different parts of the durum wheat kernel: the external bran, the intermediate layers (aleurone cells layer) and the inner endosperm (rich in starch) called ‘semolina’. The kernels are broken in three steps, i.e., passing through rifled cylinders, break rolls and reducing rolls, which are like the industrial ones and rotate with different speeds. Finally, the yielded parts are separated by sieves and sorted into three categories: the external bran layer, aleurone cells (a mix of intermediate bran layers and endosperms) and 58% of semolina (the kernel endosperm). Rapeseed and mustard seeds were smashed in different disposable tubes by Precellys Evolution Homogenizer (Bertin Technologies, France) and a greasy powder was obtained from these samples. This technology is able to lysate and homogenize different hard samples thanks to a 3D bead-beating movement that gives the same efficiency to each tube. Samples were homogenized three times for 30 s at 9000 rpm. Twenty seconds elapsed between one run of grinding and the next to prevent overheating the samples. To avoid cross-contamination from field of mustard and rapeseed, the selected common grain came from Italy, where these cultivations are absent. The purity of obtained flour was tested by an external laboratory with ELISA (RIDASCREEN^®^ FAST Senf/Mustard) and PCR assay for mustard and rapeseed identification. Reference matrices were prepared by using a round homemade mixing container with a 1070 cm^3^ capacity. First, flour was inserted inside the container as a bulk and the correct amount of powdered seeds was added to obtain two different levels of fortification: 0.1% and 0.002%. The resulting flours were then left rotating for 8 h.

### 2.3. DNA Markers Selection

Preliminary bioinformatic analysis was conducted to evaluate the most variable DNA barcoding regions that are useful for distinguishing mustard species. Nucleotide sequences of *B. nigra*, *B. juncea*, *B. napus,* and *S. alba* were obtained from NCBI Nucleotide for *rbcL*, *matK*, *psbA-trnH,* and ITS2 regions (www.ncbi.nlm.nih.gov/nucleotide/). Kimura two-parameters K2P distances for all markers were evaluated with MEGA software (Version 10.0.5) [19]. The average K2P distance, range of K2P variation and standard error for each tested marker are shown in Table S2. To have a marker of positive control for the occurrence of Brassicaceae, the *matK* locus was selected, since it has conserved regions shared among the tested Brassicaceae species that are different for *T. aestivum* (Table 1). The nuclear ITS2 region was identified as a possible candidate DNA barcode region to distinguish *S. alba* from the other three Brassica species (i.e., *B. nigra*, *B. juncea,* and *B. napus*).

**Figure 1:**
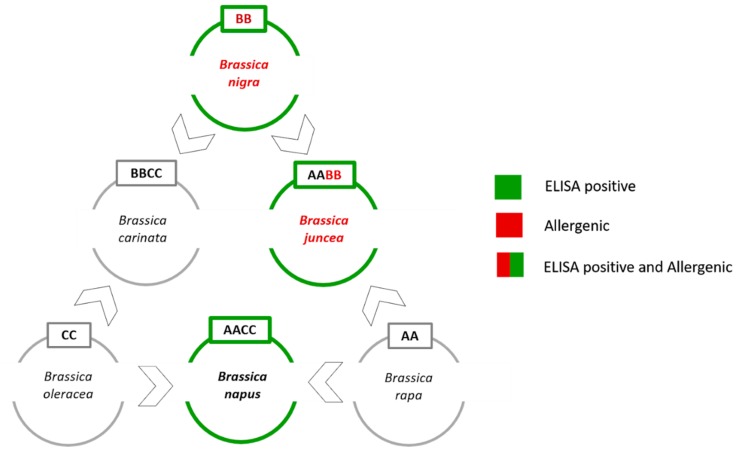
Brassica species and the U’s triangle. Three diploid species (i.e., *B. rapa*, *B. nigra*, and *B. oleracea*) which represent the AA, BB, and CC genomes are shown. The other three allotetraploid hybrid species (i.e., *B. juncea*, *B. napus*, and *B. carinata*) sharing a combination of the basic genomes (AABB, AACC, BBCC) are also reported [22].

Unfortunately, the genetic distances between some mustard species (i.e., *B. juncea* and *B. nigra)* are very small since they are members of the six species forming the so called “Brassica U’s triangle” (Figure 1) [22].

The angles of the U’s triangle are formed by three species with their own elementary genome: *B. rapa* (A-genome), *B. nigra* (B-genome), and *B. oleracea* (C-genome). The sides consist of three amphiploid species formed by the elementary genomes, *B. juncea* (AB), *B. napus* (AC), and *B. carinata* (BC). In this work, the main issue was to develop a tool to distinguish the allergenic mustard species (i.e., *B. nigra* and *B. juncea*) from the non-allergenic rapeseed (i.e., *B. napus*) that shows cross-reactivity with the commercial ELISA assays.

*B. napus* grows in crop rotation systems, and in an ideal and frequently applied rotation system, rapeseed will follow a cereal crop [23]. For this reason, rapeseed contamination in wheat crops can occur rather frequently. Conversely, *B. rapa*, *B. oleracea* and *B. carinata* grow in a different typology of fields, so the risk of contamination is low. For this reason, rapeseed is the only U’s triangle non-allergenic species that has been considered in this study.

In order to selectively distinguish each mustard species, three species-specific DNA regions were also considered (i.e., Bra019579 (Chromosome A6, 220 bp) specific for *B. juncea* and *B. napus*, pBNBH35 (Chromosome B, 280 bp) specific for *B. juncea* and *B. nigra* and Bo1g016520 (Chromosome C1, 130 bp) specific for *B. napus*). These regions were previously identified by Koh et al. [20] and Schelfhout et al. [21]. All the primer pairs used in this study are reported in Table 1.

### 2.4. Primer Design

Primer pairs listed in Table 1 were identified by bioinformatic analysis. Nucleotide sequences of *matK* region obtained from NCBI Nucleotide were aligned using ClustalW2 software (www.ebi.ac.uk/Tools/msa/clustalw2/), and a universal primer pair for Brassicaceae was de novo designed on the regions of the *matK* barcode locus mostly conserved among all the considered species and Brassicaceae family (Table 1).

Similarly, nucleotide sequences of the nuclear ITS2 locus belonging to *S. alba*, *B. nigra*, *B. juncea* and *B. napus* retrieved from Genbank NCBI were aligned using ClustalW2 and used to design a species-specific primer pair only for *S. alba* (Table 1). Both *matK* and ITS2 regions were tested with Primer–Blast tool available from NCBI (www.ncbi.nlm.nih.gov/tools/primer-blast/) to verify their specificity (i.e., Brassicaceae genus for *matK* and *S. alba* for ITS2). Similarly, primer pairs for A6, B and C1 Brassicaceae chromosomes (i.e., *B. juncea* and *B. napus* for A6, *B. juncea* and *B. nigra* for B and *B. napus* for C1) were tested. 

### 2.5. DNA Extraction and Amplification

For *T. aestivum* and the mustard and rapeseed species (Table S1), purified gDNA was obtained starting from 20 mg of seeds for each accession by using the EuroGOLD Plant DNA Mini Kit (Euroclone, Pero, Italy) following manufacturer’s instructions. Purified DNA was checked for concentration and purity by using NanoDrop™ One/OneC Microvolume UV-Vis Spectrophotometer (Thermo Fisher Scientific™, Waltham, Massachusetts, USA).

In order to evaluate the efficiency of the developed molecular tool and to guarantee its reproducibility, flour samples were analyzed following two different DNA extraction protocols. The first was the NucleoSpin^®^ Food (Macherey-Nagel GmbH, Düren, Germany), starting at 250 mg flour samples; the second was the Maxwell^®^RSC PureFood GMO and Authentication Kit (Promega, Madison, Wisconsin, USA) starting at 200 mg flour samples. For both extraction protocols, the manufacturers’ instructions were followed.

A standard PCR amplification of the selected DNA barcoding loci (*matK* and ITS2) was performed using puReTaq Ready-To-Go PCR beads (GE Healthcare Life Sciences, Italy) following the manufacturer’s instructions in a 25 μL reaction containing 1 μL 10 mM of each primer and 50 ng of gDNA. PCR cycles consisted of an initial denaturation step for 5 min at 95 °C, followed by 35 cycles of denaturation (45 s at 95 °C), annealing (45 s at different temperatures; see Table 1) and extension (1 min at 72 °C), and, hence, a final extension at 72 °C for 7 min.

For the species-specific regions (A6, B and C1) PCR was performed with the same conditions, but 2% of DMSO was added to the mix to increase the stringency of the reaction. We developed a touchdown PCR that consisted of an initial denaturation step for 5 min at 95 °C, followed by 15 denaturation cycles (45 s at 95 °C), annealing (30 s at 70 °C, this temperature is reduced by 0.7 °C every successive cycle) and extension (1 min at 72 °C), and by 14 cycles of denaturation (45 s at 95 °C), annealing (30 s at 66 °C) and extension (1 min at 72 °C) hence, a final extension at 72 °C for 7 min.

The results were interpreted as amplicons presence or absence, and were visualized using the Qiaxcel Automatic electrophoresis system (QIAGEN, Hilden, Germany). Amplicon length was measured by comparison against QX DNA Size Marker 50–800 bp v2.0. Purified amplicons were bidirectionally sequenced using an ABI 3730XL automated sequencing machine at Eurofins Genomics (Ebersberg, Germany). The 3′ and 5′ terminal portions of each sequence were clipped to generate consensus sequences for each sample. The obtained sequences were manually edited, primers were removed and were aligned pairwise. The obtained sequences were submitted to the international GenBank through the EMBL platform (see Table S1, Supplementary Materials for accession numbers). The reliability of the tested marker was assessed by adopting a standard comparison approach against a GenBank database with BLASTn (https://blast.ncbi.nlm.nih.gov/Blast.cgi) [24]. Each barcode sequence was taxonomically assigned to the plant species with the nearest matches (maximum identity > 99% and query coverage of 100%).

### 2.6. Enzyme-Linked Immunosorbent (ELISA) Assay 

Common wheat flour reference materials were also analyzed by ELISA method (RIDASCREEN^®^ FAST Senf/Mustard). This sandwich enzyme immunoassay uses antibodies specific for detecting all kinds of mustard (yellow, white, brown, black mustard). The kit declares a cross-reactivity approximately 67% with rapeseed, because of the close phylogenetic relationship. The LOD and LOQ are respectively 0.22 mg/kg and 0.5 mg/kg of mustard; the analytical workflow has been carried out in agreement with the supplier’s protocol.

## 3. Results

### 3.1. Bioinformatics Specificity Assessment of Designed Primer Pairs

Based on measurements of K2P genetic distances, the plastidial *matK* region was identified as the most universal barcode locus to signal the occurrence of Brassicaceae in food items (interspecific variation range 0.2–1.5%, average 1.01% ± 0.28%), whereas between *trnH-psbA* and ITS2, the last one was selected due to its high variability (interspecific variation range 7.0–25.0%, average 17.67% ± 2.98% for *trnH-psbA* and interspecific variation range 3.25–5.4%, average 3.95% ± 0.31% for ITS2 ), and wide taxonomic coverage in NCBI GenBank. Unfortunately for *trnH-psbA,* only a few sequences were available in public databases, thus impeding an extensive taxonomic survey on this region.

Both *matK* and ITS2 were tested with the Primer–Blast tool available from NCBI (www.ncbi.nlm.nih.gov/tools/primer-blast/) to verify both primer pair specificity and a possible cross-amplification with *T. aestivum*. In silico PCR results confirmed specificity for Brassicaceae genus for *matK* primer pair and *S. alba* for ITS2 primer pair. Both primer pairs showed no amplification for *T. aestivum*. Concerning the A6, B, and C1 loci, in silico PCR results suggested that the locus A6 is specific for *B. juncea* and *B. napus*, locus B is specific for *B. juncea* and *B. nigra,* and locus C1 is specific for *B. napus*. Finally, they were also tested to verify a possible cross-amplification with *T. aestivum*. A6 and C1 primer pairs showed a possible poisitive amplification with 4 mismatches, while B primer pair did not amplify *T. aestivum.*

### 3.2. Primer Pairs Testing on Plant Dataset

All the selected DNA barcoding markers and those found in Kho [20] and Schelfhout [21] were tested on all 12 plant samples tested. DNA extraction was successful for all 12 plant samples (see Table S1) with high DNA quality and good yield (i.e., 30–40 ng/μL). Concerning the amplification step, *matK* and ITS2 yielded a single band on electrophoresis and exhibited 100% amplification success with the designed primer pairs (Table 2).

All the PCR products were visualized using the Qiaxcel Automatic electrophoresis system (Figure S1), and amplicon length for *matK* was 280 bp, while in the case of ITS2 it was 220 bp. Primer pairs efficacy was also validated by Sanger sequencing and comparison against reference molecular databases. All amplicons were sequenced and high-quality bidirectional sequences were obtained. The sequences were compared against the NCBI GenBank database and the obtained results confirmed primer pairs specificity (Brassicaceae genus for *matK* and *S. alba* for ITS2), showing a 100% identity to the target species with BLASTn analysis.

The specificity of the amplification of the three loci identified by Kho and collaborators [20] and Schelfhout and collaborators [21] on the specific Brassicaceae samples was also tested. Concerning the amplification, A6, B, and C1 loci yielded a single band on electrophoresis and exhibited 100% amplification success with specific primer pairs (Table 2). All the PCR products were visualized using the Qiaxcel Automatic electrophoresis system and amplicon length for A6 loci was 220 bp, for B loci was 280 bp, while C loci was 130 bp. High-quality bidirectional sequences were obtained from the amplified regions. The amplified regions were compared with NCBI GenBank. Results confirmed all primer pair specificities (i.e., *B. juncea* and *B. napus* for A6, *B. juncea* and *B. nigra* for B, and *B. napus* for C1), showing a 100% identity to the target species with BLASTn analysis [20,21].

### 3.3. Primer Pairs Testing on Contaminated Flours

To evaluate the efficacy of the method for detecting mustard allergenic ingredients in wheat flour, the analysis was focused on two DNA barcoding markers and the three species-specific loci A6, B, and C1 on pure and artificially contaminated flour samples. DNA extraction was successful for all 7 flour samples (Table 3) with high DNA quality and good yield (i.e., 30–40 ng/μL). Results suggested that all the tested primer pairs were sensitive enough to identify contaminant DNA in the tested flours; however, A6 and C1 produced several aspecific amplicons. Considering that the B primer pair represents the most reliable tool for identifing the occurrence of *B. juncea* and *B. nigra*, we tested only its efficacy on the contaminated flour samples. The results provided in Table 3 show that all three selected markers (i.e., *matK*, ITS2 and B) are very sensitive at the lowest (i.e., 0.002% of mustard) level of contamination. In all cases, none of the primer pairs amplified *T. aestivum*.

### 3.4. DNA Analysis vs. ELISA Tests

The same flour samples (contaminated and uncontaminated, Table 3) were also analysed for mustard detection by ELISA approach using a commercially available kit, in agreement with previously reported data (Table 3). Compared to the ELISA assay, the DNA-based innovative approach presented here confirms the target detection of the occurrence of mustard; conversely, in samples contaminated by *B. napus,* it shows amplification only with *matK* primer pair.

## 4. Discussion and Conclusions

In this study, the usefulness of DNA barcoding as a tool to verify the possible contamination of wheat flours by mustard was investigated. This is one of the cases known as “hidden allergens” [25], because these inadvertently added contaminants are not mentioned on packaging, and most consumers may not be aware of being allergic to these products. In the case of mustard species, the 2S albumin stored in the seeds show a high allergenic power [26,27]. This is a high heat-resistance protein, and as such, it could remain in its intact form during high temperature industrial processes [28]. The DNA barcoding analysis confirmed that *T. aestivum* is clearly recognizable from the members belonging to Brassicaceae investigated in this study (i.e., *S. alba, B. nigra, B. juncea,* and *B. napus*) at both the chloroplast barcode *matK* and the nuclear ITS2 markers. The first important result of this work is the identification of two specific primer pairs targeting the two DNA barcoding loci able to distinguish the common wheat flour from the contaminated one, by non-allergenic compound (i.e., rapeseed from the field) and allergenic compound (i.e. mustard).

Concerning mustard contaminants, *S. alba* can be easily distinguished from *B. nigra*, *B. juncea,* and *B. napus* using the ITS2 DNA barcoding species-specific primer pair. However, neither of the DNA barcoding regions (i.e., *matK* and ITS2) were able to distinguish *B. napus* from the allergenic species *B. juncea*; therefore, the primer pair for the B locus is necessary to better distinguish the allergenic species from the non-allergenic *B. napus*. It is important to highlight that the main criticism of this diagnostic system is related to the phylogenetic position of the considered taxa [22]. Therefore, the B primer pairs on B genome of triangle were selected to detect *B. juncea* and *B. nigra* and exclude *B. napus*.

**Figure 2:**
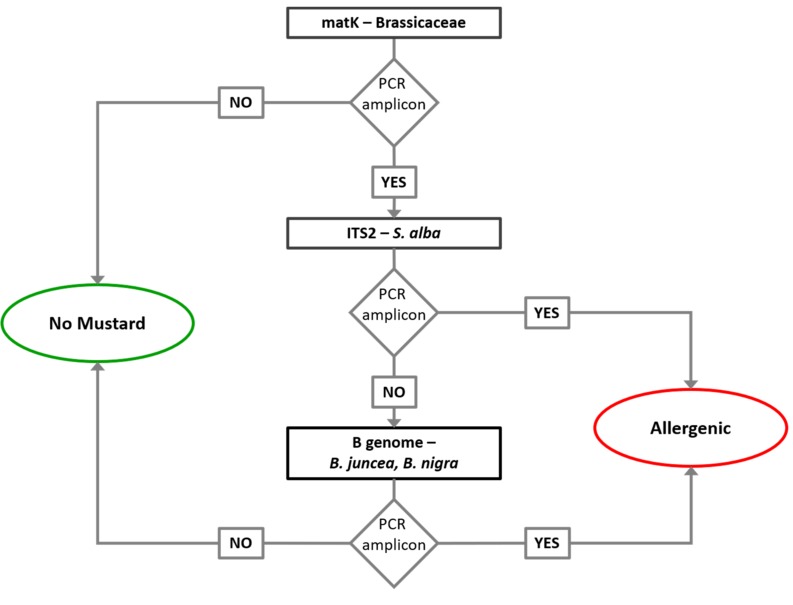
Analytical workflow for mustard detection in wheat flour.

Based on the obtained results, a short analytical pipeline to perform quality control on wheat flours was developed. A simplified DNA analytical process (Figure 2) consisting of the *matK* DNA barcoding amplification with specific primers to identify the occurrence of Brassicaceae DNA in flour and ITS2 amplification with specific primer to identify possible contamination with *S. alba* was proposed. In the case of the positive result for *matK*, to exclude the *B. napus* contamination, B analysis was also performed. The amplification of this locus is sufficient to verify the occurrence of allergenic *Brassica* spp. characterized by B genome. The developed tests on DNA markers showed high sensitivity when compared to ELISA immunoassay and could be routinely adopted to integrate the conventional ELISA and to exclude the false positive cases of this due to cross-reactions with *B. napus*.

In conclusion, one of the main goals of molecular diagnostics is the simplification of analytical systems, and DNA barcoding offered this opportunity. Several authors propose screening species-specific loci to detect black mustard or brown mustard in food items [29]. However, each of these loci provides the identification of a specific marker for only one species, and this involves several analyses for each food product. Conversely, the analytical approach proposed here relies on a universal system to clarify the purity of a food matrix or to identify non-allergenic contaminating species. Only in case of positivity for allergenic species will it be possible to characterize specific DNA loci to precisely identify the occurring allergenic species.

Another advantage of the DNA barcoding approach is that it is more versatile than other diagnostic tools, and its adoption could open opportunities for a precise screening not only for specialized laboratories, but also, in the near future, to consumers. In a previous work, Valentini and co-workers developed NanoTracer [30], a rapid molecular authentication tool based on a simple PCR reaction of the barcode or genome-specific loci that displays high interspecies genetic divergence. Species-specific primers can be easily coupled with the high sensitivity of nanotechnologies to provide rapid and user-friendly colorimetric systems to achieve food authentication [30]. These kinds of innovations will make molecular-based identification affordable to non-specialized personnel, such as small companies where high-tech laboratory facilities are not available. 

## Figures and Tables

**Table 1:** List of primers used for DNA analysis. The table includes primer specificity, marker region, annealing temperature (Ta) based on experimental analysis, amplicon length (bp), and references.

Primer Name	5′-3′	Species Specificity	Marker Region	Ta (Annealing Temperature)	Amplicon Length (bp)	References
Bras_matK F	CTACGCAAGCAGTCTTCTCATT	Brassicaceae	*matK*	60 °C	280	This study
Bras_matK R	TTGCGATTGAAACCATACGGA					
S.alba_ITS2 F	CAGAATCCCGTGAACCATCGAGTC	*Sinapis alba*	ITS2	64 °C	220	This study
S.alba_ITS2 R	GACAATATGACGAGGTTACAA					
A6 F	CCAGCGAAGGATTTGACGAC	*Brassica juncea, Brassica napus*	*Bra019579*	63 °C	220	[20]
A6 R	GACGAATCGAGTGCCCTG					
B F	GGCATCTGAAGAGAGAGTCCCTTTG	*Brassica juncea, Brassica nigra*	*pBNBH35*	63 °C	280	[21]
B R	ATCTTCTTCTTGCCATGAGTGGCC					
C1 F	TGCTGCGCCGAACAATAG	*Brassica napus*	*Bo1g016520*	60 °C	130	[20]
C1 R	CCGATCGTGGTTCATATTGC					

**Table 2:** Amplicon PCR results on seed samples. Successful PCR amplification is indicated by “x”, failure is indicated by “-”. *matK* primers are specific for Brassicaceae and amplify all the tested samples, ITS2 primer pair is specific for *S. alba*, A6 primer pair is specific for *B. juncea* and *B. napus*, B primer pair is specific for *B. juncea* and *B. nigra,* and C1 primer pair is specific for *B. napus*.

Sample ID	*matK*	ITS2	A6	B	C1
Bni_01	x	-	-	x	-
Bni_02	x	-	-	x	-
Bni_03	x	-	-	x	-
Bju_01	x	-	x	x	-
Bju_02	x	-	x	x	-
Bju_03	x	-	x	x	-
Bna_01	x	-	x	-	x
Bna_02	x	-	x	-	x
Bna_03	x	-	x	-	x
Sal_01	x	x	-	-	-
Sal_02	x	x	-	-	-
Sal_03	x	x	-	-	-

**Table 3:** List of flour samples, before and after contamination, with details concerning the species of contaminants and their concentrations (For this study we considered only species from Europe) and PCR and ELISA test results. Successful PCR amplification is indicated by “x”, failure is indicated by “-”. Concerning PCR analysis, *matK* is specific for Brassicaceae, ITS2 for *Sinapis alba*, and B for *Brassica nigra* and *Brassica juncea*. ELISA test is specific for S2 albumin (i.e., *Brassica juncea*, *Brassica nigra,* and *Sinapis alba*) with a cross-reactivity approximately 67% with *Brassica napus*.

Samples ID	Herbal Contamination	Herbal Concentration	*matK*	ITS2	B	ELISA Results	ELISA Results mg/kg
Flour uncontaminated	*/*	/	-	-	-	-	1.4
Flour_01_A	*Brassica nigra, Brassica juncea, Sinapis alba*	0.1%	x	x	x	x	610
Flour_01_B	*Brassica nigra, Brassica juncea, Sinapis alba, Brassica napus*	0.1%	x	x	x	x	1300
Flour_01_C	*Brassica napus*	0.1%	x	-	-	x	1200
Flour_002_A	*Brassica nigra, Brassica juncea, Sinapis alba*	0.002%	x	x	x	x	43
Flour_002_B	*Brassica juncea, Sinapis alba, Brassica napus*	0.002%	x	x	x	x	26
Flour_002_C	*Brassica napus*	0.002%	x	-	-	x	49

ELISA: Enzyme-Linked Immunosorbent; PCR: Polymerase Chain Reaction

## Supplementary Materials

The following are available online at https://www.mdpi.com/2073-4425/10/3/234/s1, Table S1: List of the analysed samples with details concerning the geographical origins, the CGN accession numbers, the GenBank Accession Number, and the specimen voucher, Table S2. Percentages of the average Kimura two-parameter (K2) distance, range of K2P mean variation, standard error (SE), and range for each tested marker, Figure S1: Amplification products obtained by the set of primers considered in this work.

## References

[1] Regulation (EU) No. 1169/2011 of the European Parliament and of the Council of 25 October 2011. https://eur-lex.europa.eu/legal-content/EN/TXT/PDF/?uri=CELEX:32011R1169&from=EN.

[2] Taylor S.L., Hefle S.L. (2006). Food allergen labeling in the USA and Europe. Curr. Opin. Allergy Clin. Immunol..

[3] Fiocchi A., Dahdah L., Fierro V., Artesani M.C., Valluzzi R. (2018). Food allergy trends at the crossing among socio-economics, history and geography. Curr. Opin. Allergy Clin. Immunol..

[4] Dhondalay G.K., Rael E., Acharya S., Zhang W., Sampath V., Galli S.J., Tibshirani R., Boyd S.D., Maecker H., Nadeau K.C. (2018). Food allergy and omics. J. Allergy Clin. Immunol..

[5] Graziano S., Gullì M., Marmiroli N. (2018). Detection of allergen coding sequences of kiwi, peach, and apple in processed food by qPCR. J. Sci. Food Agric..

[6] Eischeid A.C., Stadig S.R. (2018). A group-specific, quantitative real-time PCR assay for detection of crab, a crustacean shellfish allergen, in complex food matrices. Food Chem..

[7] Galimberti A., de Mattia F., Losa A., Bruni I., Federici S., Casiraghi M., Martellos S., Labra M. (2013). DNA barcoding as a new tool for food traceability. Food Res. Int..

[8] Galimberti A., Bruno A., Mezzasalma V., de Mattia F., Bruni I., Labra M. (2015). Emerging DNA-based technologies to characterize food ecosystems. Food Res. Int..

[9] Handy S.M., Deeds J.R., Ivanova N.V., Hebert P.D.N., Hanner R.H., Ormos A., Weigt L.A., Moore M.M., Yancy H.F. (2011). A single-laboratory validated method for the generation of DNA barcodes for the identification of fish for regulatory compliance. J. AOAC Int..

[10] Barcaccia G., Lucchin M., Cassandro M. (2016). DNA Barcoding as a Molecular Tool to Track Down Mislabeling and Food Piracy. Diversity.

[11] Federici S., Galimberti A., Bartolucci F., Bruni I., Mattia F.D., Cortis P., Labra M. (2013). DNA barcoding to analyse taxonomically complex groups in plants: The case of Thymus (Lamiaceae). Bot. J. Linn. Soc..

[12] Barbuto M., Galimberti A., Ferri E., Labra M., Malandra R., Galli P., Casiraghi M. (2010). DNA barcoding reveals fraudulent substitutions in shark seafood products: The Italian case of “palombo” (*Mustelus* spp.). Food Res. Int..

[13] Sharma A., Verma A.K., Gupta R.K., Dwivedi P.D. (2017). A Comprehensive Review on Mustard-Induced Allergy and Implications for Human Health. Clin. Rev. Allergy Immunol..

[14] Menéndez-Arias L., Domínguez J., Moneo I., Rodríguez R. (1990). Epitope mapping of the major allergen from yellow mustard seeds, Sin a I. Mol. Immunol..

[15] Cuhra P., Gabrovská D., Rysová J., Hanák P., Stumr F. (2011). ELISA kit for mustard protein determination: Interlaboratory study. J. AOAC Int..

[16] Gylling H. (2006). Rapeseed oil does not cause allergic reactions. J. Allergy.

[17] Fiocchi A., Dahdah L., Riccardi C., Mazzina O., Fierro V. (2016). Preacutionary labelling of cross-reactive foods: The case of rapeseed. Asthma Res. Pract..

[18] Sun R. (2015). The Brassica rapa Genome.

[19] Tamura K., Dudley J., Nei M., Kumar S. (2007). MEGA4: Molecular evolutionary genetics analysis (MEGA) software version 4.0. Mol. Biol. Evol..

[20] Koh J.C.O., Barbulescu D.M., Norton S., Redden B., Salisbury P.A., Kaur S., Cogan N., Slater A.T. (2017). A multiplex PCR for rapid identification of Brassica species in the triangle of U. Plant Methods.

[21] Schelfhout C.J., Snowdon R., Cowling W.A., Wroth J.M. (2004). A PCR based B-genome-specific marker in Brassica species. Theor. Appl. Genet..

[22] Nagaharu U. (1935). Genome analysis in Brassica with special reference to the experimental formation of *B. napus* and peculiar mode of fertilization. Jpn J. Bot..

[23] Hegewald H., Wensch-Dorendorf M., Sieling K., Christen O. (2018). Impacts of break crops and crop rotations on oilseed rape productivity: A review. Eur. J. Agron..

[24] Altschul S.F., Gish W., Miller W., Myers E.W., Lipman D.J. (1990). Basic local alignment search tool. J. Mol. Biol..

[25] Baker M.G., Saf S., Tsuang A., Nowak-Wegrzyn A. (2018). Hidden allergens in food allergy. Ann. Allergy Asthma Immunol..

[26] Radauer C., Kleine-Tebbe J., Beyer K., Kleine-Tebbe J., Jakob T. (2017). Stable Plant Food Allergens II: Storage Proteins. Molecular Allergy Diagnostics: Innovation for a Better Patient Management.

[27] Moreno F.J., Clemente A. (2008). 2S Albumin Storage Proteins: What Makes them Food Allergens?. Open Biochem. J..

[28] Sirvent S., Palomares O., Cuesta-Herranz J., Villalba M., Rodríguez R. (2012). Analysis of the structural and immunological stability of 2S albumin, nonspecific lipid transfer protein, and profilin allergens from mustard seeds. J. Agric. Food Chem..

[29] Palle-Reisch M., Wolny M., Cichna-Markl M., Hochegger R. (2013). Development and validation of a real-time PCR method for the simultaneous detection of black mustard (*Brassica nigra*) and brown mustard (*Brassica juncea*) in food. Food Chem..

[30] Valentini P., Galimberti A., Mezzasalma V., De Mattia F., Casiraghi M., Labra M., Pompa P.P. (2017). DNA barcoding meets nanotechnology: Development of a universal colorimetric test for food authentication. Angew. Chem. Int. Ed. Engl..

