# Supplementary material for: Development of a DNA Barcoding-Like Approach to Detect Mustard Allergens in Wheat Flours
Source: Genes (Basel). 2019 Mar 19;10(3):234. doi: 10.3390/genes10030234 (PMC6470546; doi:10.3390/genes10030234)
Supplement: Supplementary file 1 [file genes-10-00234-s001.pdf]

genes MDPI

Supplementary Materials

Table S1: List of the analysed samples with details concerning the geographical origins, the CGN accession numbers, the GenBank Accession Number and the specimen voucher.

Taxa	Origin	CGN_Accession number	GenBank Accession Number	Specimen voucher
Brassica nigra	Germany	CGN06624	LR217828 (*matK*); LR217834 (B)	Bni_01
	Greek	CGN06619	LR217829 (*matK*); LR217835 (B)	Bni_02
	Germany	CGN06620	LR217830 (*matK*); LR217836 (B)	Bni_03
Brassica juncea	Germany	CGN06616	LR217822 (*matK*); LR217843 (A6); LR217837 (B)	Bju_01
	Sweden	CGN12009	LR217823 (*matK*); LR217844 (A6), LR217838 (B)	Bju_02
	Germany	CGN06615	LR217824 (*matK*); LR217845 (A6); LR217839 (B)	Bju_03
Brassica napus	Netherlands	CGN13917	LR217825 (*matK*); LR217846 (A6); LR217840 (C1)	Bna_01
	France	CGN14113	LR217826 (*matK*); LR217847 (A6); LR217841 (C1)	Bna_02
	Germany	CGN12014	LR217827 (*matK*); LR217848 (A6); LR217842 (C1)	Bna_03
Sinapis alba	Italy	CGN11981	LR217831 (*matK*); LR217819 (ITS2)	Sal_01
	Sweden	CGN06662	LR217832 (*matK*); LR217820 (ITS2)	Sal_02
	Germany	CGN11969	LR217833 (*matK*); LR217821 (ITS2)	Sal_03

Table S2. Percentages of the average Kimura two-parameter (K2) distance, range of K2P distances, standard error (SE), and range for each tested marker. Overall mean distances and intraspecific and interspecific comparisons are indicated.

	Intraspecific comparison		Interspecific comparison	
Locus	Mean % variation (SE%)	Range (%)	Mean % variation (SE%)	Range (%)
rbcL	0.75 (0.1)	0-3.0	1.05 (0.29)	0-1.8
matK	0 (0)	0	1.01 (0.28)	0.2-1.5
psbA-trnH	4.6 (1.66)	1.0-8.0	17.67 (2.98)	7.0-25.0
ITS2	1.5 (0.75)	0-3.0	3.95 (0.31)	3.25-5.4

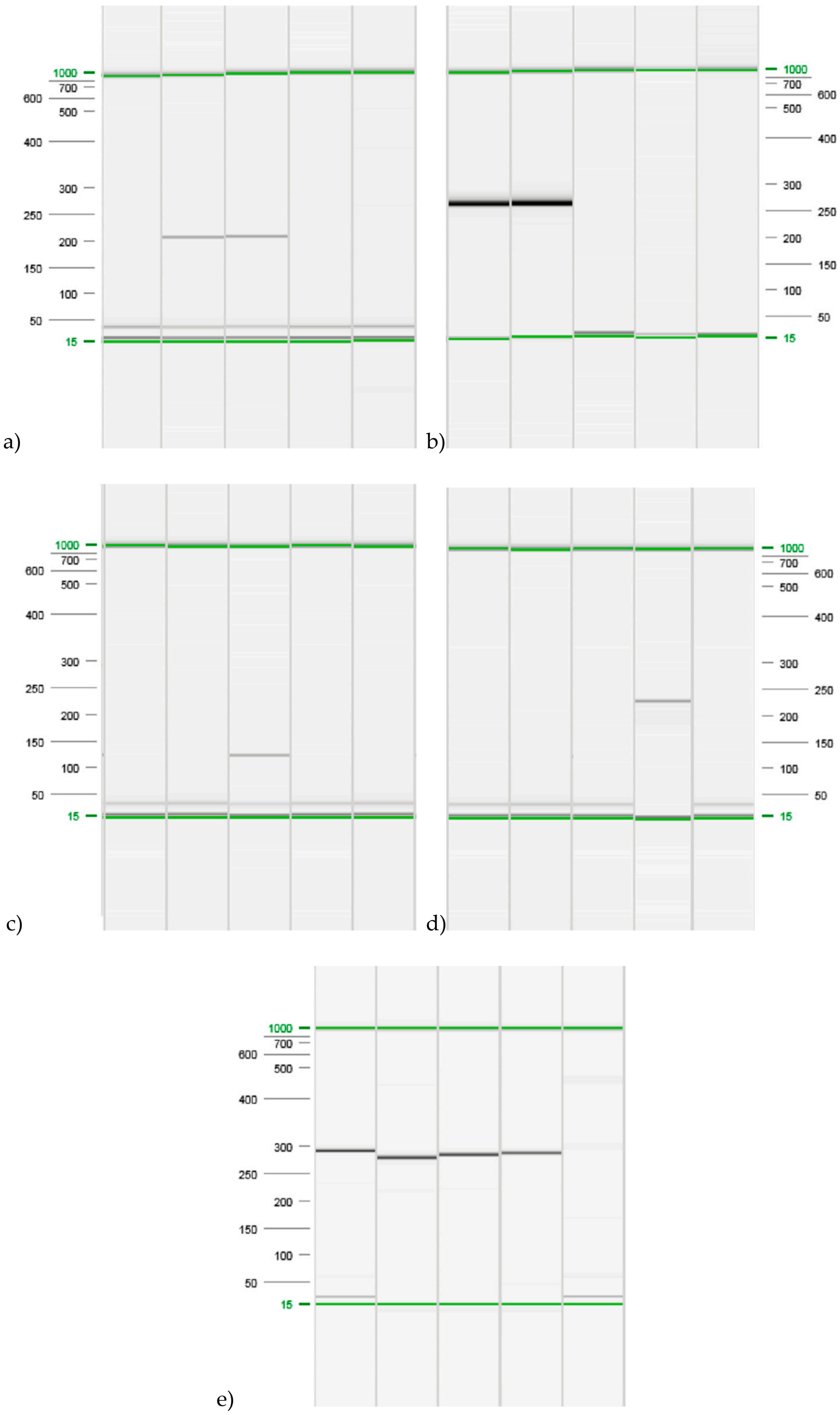

Figure S1: Amplification products obtained by the set of primers considered in this work. a) Primer A6: Lane 1: *Brassica nigra*, lane 2: *Brassica juncea*, lane 3: *Brassica napus*, lane 4: *Sinapis alba*, lane 4: Negative control; b) Primer B: Lane 1: *Brassica nigra*, lane 2: *Brassica juncea*, lane 3: *Brassica napus*, lane 4: *Sinapis alba*, lane 4: Negative control; c) Primer C1 : Lane 1: *Brassica nigra*, lane 2: *Brassica juncea*, lane 3: *Brassica napus*, lane 4: *Sinapis alba*, lane 4: Negative control; d) Primer S.alba_ITS2: : Lane 1: *Brassica nigra*, lane 2: *Brassica juncea*, lane 3: *Brassica napus*, lane 4: *Sinapis alba*, lane 4: Negative control; e) Primer Bras_matK: : Lane 1: *Brassica nigra*, lane 2: *Brassica juncea*, lane 3: *Brassica napus*, lane 4: *Sinapis alba*, lane 4: Negative control.
